# Conversion Discriminative Analysis on Mild Cognitive Impairment Using Multiple Cortical Features from MR Images

**DOI:** 10.3389/fnagi.2017.00146

**Published:** 2017-05-18

**Authors:** Shengwen Guo, Chunren Lai, Congling Wu, Guiyin Cen, The Alzheimer's Disease Neuroimaging Initiative, Michael W. Weiner

**Affiliations:** ^1^Department of Biomedical Engineering, South China University of Technology Guangzhou, China; ^2^Guangdong General Hospital Guangzhou, China

**Keywords:** mild cognitive impairment, conversion, cortical feature, sparse-constrained regression, feature reduction, classification

## Abstract

Neuroimaging measurements derived from magnetic resonance imaging provide important information required for detecting changes related to the progression of mild cognitive impairment (MCI). Cortical features and changes play a crucial role in revealing unique anatomical patterns of brain regions, and further differentiate MCI patients from normal states. Four cortical features, namely, gray matter volume, cortical thickness, surface area, and mean curvature, were explored for discriminative analysis among three groups including the stable MCI (sMCI), the converted MCI (cMCI), and the normal control (NC) groups. In this study, 158 subjects (72 NC, 46 sMCI, and 40 cMCI) were selected from the Alzheimer's Disease Neuroimaging Initiative. A sparse-constrained regression model based on the l2-1-norm was introduced to reduce the feature dimensionality and retrieve essential features for the discrimination of the three groups by using a support vector machine (SVM). An optimized strategy of feature addition based on the weight of each feature was adopted for the SVM classifier in order to achieve the best classification performance. The baseline cortical features combined with the longitudinal measurements for 2 years of follow-up data yielded prominent classification results. In particular, the cortical thickness produced a classification with 98.84% accuracy, 97.5% sensitivity, and 100% specificity for the sMCI–cMCI comparison; 92.37% accuracy, 84.78% sensitivity, and 97.22% specificity for the cMCI–NC comparison; and 93.75% accuracy, 92.5% sensitivity, and 94.44% specificity for the sMCI–NC comparison. The best performances obtained by the SVM classifier using the essential features were 5–40% more than those using all of the retained features. The feasibility of the cortical features for the recognition of anatomical patterns was certified; thus, the proposed method has the potential to improve the clinical diagnosis of sub-types of MCI and predict the risk of its conversion to Alzheimer's disease.

## Introduction

The most common form of dementia, Alzheimer's disease (AD), is a progressive neurodegenerative disorder in the elderly that affects their health and quality of life; it affects memory, thinking, behavior, and the ability to perform everyday activities in the elderly worldwide (Kandimalla et al., 2011; Zhang et al., 2014). According to the 2013, 2014, and 2015 World Alzheimer Report (Prince et al., 2013, 2014, 2015), approximately 35.6 million people were suffering from dementia in 2010, and the worldwide cost of dementia care was greater than US$ 600 billion or approximately 1% of the global GDP. It is estimated that this number will reach 65.7 million in 2030 and 115.4 million in 2050 owing to the dramatic increase in the global aging population. Unfortunately, the reasons for, and mechanisms and progression of AD are yet to be fully understood; there are no established diagnostic biomarkers and effective treatments for dementia (Chételat et al., 2010). Prior studies have suggested that the pathologic timeline of AD may begin anywhere from a few years to decades before clinical diagnosis, with an initial asymptomatic phase (preclinical AD) followed by a phase with MCI (Jack et al., 2010; Hinrichs et al., 2011). Therefore, the prevention and detection of dementia in its early stage and the tracking and prediction of its progress are critical to preventing the onset of this disease or slowing the advancement of neurodegeneration.

As is well known, MCI is considered as a transitional stage between normal aging and the development of AD (Petersen et al., 2009), and patients with MCI, especially amnestic MCI, have a high risk (10–15%) of converting to AD patients (Petersen et al., 1999; Morris et al., 2001; Morris and Cummings, 2005). Therefore, it is very important to investigate the progression and development of MCI, in order to reveal subtle changes in brain structure and elucidate dysfunction in cognition. It is becoming increasingly important to seek effective biomarkers to monitor MCI development, track its progress, and predict its conversion to AD at an early stage.

The existing biomarkers for MCI detection and prediction of development to AD can be divided into three categories: gene biomarkers, chemical biomarkers, and neuroimaging biomarkers. The ε 4 allele of the human apolipoprotein E gene (APOE) is a well-proven genetic risk factor associated with brain integrity and can be used as a biomarker for detecting the early stages of MCI (Farlow et al., 2004; Risacher et al., 2013). Single nucleotide polymorphisms (SNPs) in the APOE and the TOMM40, EPHA4, TP63, and NXPH1 genes were confirmed as markers strongly associated with the destruction of synaptic integrity in MCI and AD patients (Shen et al., 2010). Furthermore, the sortilin-related receptor gene (SORL1) showed potential as a risk factor for MCI conversion to AD (Piscopo et al., 2015).

The use of chemical biomarkers has also been widely explored; these include the use of cerebrospinal fluid (CSF) Aβ, CSF tau (Mattsson et al., 2012; Kandimalla et al., 2013; Ferreira et al., 2014), 11C-Pittsburgh compound B positron emission tomography (11C PIB-PET), Aβ imaging (Mattsson et al., 2012; Banzo et al., 2015), FDG-PET (Mosconi et al., 2009; Zhang et al., 2012; Smailagic et al., 2015), magnetic resonance spectroscopy (MRS), and magnetic resonance spectroscopy imaging (MRSI) (Modrego et al., 2005, 2011; Pilatus et al., 2009; Targosz-Gajniak et al., 2013; Gao and Barker, 2014; Watanabe et al., 2015). It has been demonstrated that changes in chemical biomarkers have the potential to reveal progressive accumulations of MCI and AD pathologies.

Neuroimaging measurements, including structural magnetic resonance imaging (sMRI) (Rose et al., 2000; Kim et al., 2012; Franko and Joly, 2013; Boutet et al., 2014; Peng et al., 2015), functional magnetic resonance imaging (fMRI) (Singh et al., 2012; Li et al., 2015), have become the mainstay in MCI and AD diagnosis and progressive risk evaluation owing to their visual representation, intuitive illustration, and reliability.

The pattern of cortical atrophy obtained from sMRIs and morphological analysis have been extensively investigated to discriminate the MCI, the AD from the normal elderly or normal controls (NCs) (Fan et al., 2008; Schuff et al., 2009; Franko and Joly, 2013; Boutet et al., 2014; Peng et al., 2015), distinguish the stable MCI (sMCI) from the converted MCI (cMCI) (Chetelat et al., 2005; Davatzikos et al., 2011), and trace and predict conversion from NCs to MCI and from the MCI to AD (Driscoll et al., 2009; Davatzikos et al., 2011; Adaszewski et al., 2013; Eskildsen et al., 2013b). The most commonly used sMRI biomarker in these studies is the hippocampal volume, which is related to memory and spatial navigation. Some recent works incorporate the hippocampus with other dominant regions including the parahippocampus, the inferior temporal gyrus, the middle temporal gyrus, and the posterior cingulate cortex as sMRI biomarkers. In addition to decreases in gray matter (GM) volume, other morphological signatures such as the thickness of GM, depth and surface area of the sulus, and curvatures of the sulus surface have been of central interest in characterizing cortical atrophy and change. For example, with disease progression from NC to MCI and from MCI to AD, the mean curvature decreased and the sulcal depth tended to be shallower; in addition, the most remarkable sulcal widening was observed in the temporal lobe (Im et al., 2008). The cortical thinning areas were related to hippocampal atrophy (Kim et al., 2012). The cortical thickness could be considered as a quantitative biomarker of dementia (Dickerson et al., 2012), and the cortical thickness and the sulcal depth were adopted as biomarkers for early detection of AD and prediction of conversion (Park et al., 2013). Based on the observations of hippocampal atrophy and glucose hypometabolism of strongly connected regions including the posterior cingulate, the thalamus, the parahippocampal gyrus, and the hippocampus, GM atrophy appears to result in a subsequent degeneration in white matter (WM) (Villain et al., 2008). Magnetic resonance (MR) diffusion tensor imaging (DTI) revealed AD-related abnormalities in the structural integrity of WM such as corpus callosum, superior longitudinal fasciculus, and cingulum (Rose et al., 2000; Medina et al., 2006). Recent studies suggest that the MCI and AD groups have significant disruption in either the structural network or functional network (Dickerson and Sperling, 2009; Dai and He, 2014; Li et al., 2014, 2015; Wang et al., 2015; Yi et al., 2015); these profiles indicated that aberrant network dysfunctions might be the potential biomarkers or predictors of MCI progression.

Because these three types of biomarkers complement each other, there are an increasing number of studies being conducted to combine multiple biomarkers in order to improve the degeneration identification and conversion prediction of MCI and AD cohorts. For example, MRI morphometry, CSF biomarkers, and neuropsychological and functional measurements were combined as features to predict MCI conversion with an accuracy of 67.13%, a sensitivity of 96.43%, and a specificity of 48.28% (Cui et al., 2011). Using genetic, structural, and functional imaging biomarkers, the support vector machine (SVM) classifier attained an accuracy of 64.57%, a sensitivity of 72.20%, and a specificity of 58.90% (Singh et al., 2012). More studies have been focused on attempting to evaluate the combined role of different biomarkers such as CSF and MRI markers (Davatzikos et al., 2011; Westman et al., 2012), 18 F-FDG and 11C-PIB-PET (Zhang et al., 2012), MRI measures, PET-FDG numerical summary, CSF biomarkers (t-tau, p-tau, and Aβ42), and APOE genotype (Kohannim et al., 2010).

These combination studies made use of the multivariate model to combine different measures to construct classifiers to improve the prediction of AD dementia in patients with MCI. However, the main unresolved challenge is how to effectively integrate various measures for classification and identification of the dominant features associated with MCI conversion. Traditional filter-based feature selection methods such as *F*-statistic and *t*-test focus on the relation of a feature with respect to the class label distribution of the data, but they do not take into account the correlation of features. Principle component analysis (PCA), a well-known dimensionality reduction method, involves an orthogonal transformation to convert a set of observations of possibly correlated variables into a set of values of linearly uncorrelated variables called principal components. All of the principal components are orthogonal to each other and, as a whole, form an orthogonal basis for the space of the original data. Although the PCA reveals the internal structure of the data in an effective manner that projects the entire set of data onto a different subspace, it experiences difficulty in comparing the significance of different features and understanding the causes of dementia and its development in pathology. In addition to the statistic method and PCA, embedded methods such as correlation-based feature selection (Hall and Smith, 1999) and the SVM recursive feature elimination (SVM-RFE) (Guyon et al., 2002) considers feature selection in the training process. Although the SVM-RFE can ensure high performance, it is a computationally expensive task.

Recently, the theory of sparse representation in dimensionality reduction has been widely investigated and successfully integrated with compressed sensing and other applications (Nie et al., 2010; Wang et al., 2011; Bao et al., 2013; Yang et al., 2013; Yan and Yang, 2015). This optimization problem, which uses sparsity constraints, attempts to provide a sparse vector whose consistency with the acquired data is usually determined based on the squared error. The subspace learning and dimensionality selecting strategy is preferable in regression and classification problems in multiple-biomarker analysis on neurodegenerative disorders such as MCI/AD identification, sMCI/cMCI discrimination, and MCI conversion prediction.

Most reports suggest that subjects with MCI exhibit atrophy in cortical association areas with prominent involvement of the temporal, parietal, and frontal regions, and cortical measurements and its changes are the significant biomarkers for detecting the progression of MCI (McEvoy et al., 2011; Li et al., 2012). Nonetheless, it is still difficult to systematically explore an effective dimensionality reduction strategy and perform discrimination analysis of cortical surface features from baseline and longitudinal MR images for the NC, sMCI, and cMCI groups. In this study, we calculated four types of cortical surface features from 2 years of follow-up MR images, then combined the baseline features with longitudinal measures, and used dimensionality reduction based on sparse representation to retrieve the dominant features for the SVM classification of the three compared groups.

## Materials and methods

### Subjects

One hundred and fifty-eight subjects in this study were selected from the Alzheimer's Disease Neuroimaging Initiative (ADNI) database (http://www.loni.ucla.edu/ADNI/). This study included 72 NC subjects, 46 sMCI subjects, and 40 cMCI subjects. The MR image data for each subject was acquired and evaluated using two cognitive impairment measures—the mini-mental state examination (MMSE) and clinical dementia rating (CDR)—at three time points: baseline, 12, and 24 months. Among the identified MCI patients at baseline, an individual was determined as a patient with cMCI if the subject had converted to AD within 2 years, and the rest were considered as sMCI subjects. Table 1 shows the subject demographics and dementia status.

**Table 1:** **Subject demographics and dementia status in baseline and 12, 24 months**.

**Group**	**Sample Size**	**Gender (M/F)**	**Age (Years)**	**MMSE**			**CDR**		
				**Baseline**	**12 months**	**24 months**	**Baseline**	**12 months**	**24 months**
NC	72	38/34	76.06 ± 5.61 (59.9~89.6)	29.16 ± 0.94 (26~30)	28.97 ± 1.41 (24~30)	29.22 ± 0.97 (27~30)	0.02 ± 0.10 (0~0.5)	0.07 ± 0.32 (0~2)	0.20 ± 0.53 (0~4)
sMCI	40	24/16	74.58 ± 6.60 (61~86.4)	27.26 ± 1.76 (24~30)	27.43 ± 2.14 (23~30)	26.93 ± 2.49 (22~30)	1.39 ± 0.57 (0.5~2.5)	1.77 ± 1.00 (0.5~5)	2.08 ± 1.41 (0.5~7)
cMCI	46	34/12	74 ± 7.36 (55.2~87.7)	26.38 ± 1.77 (24~30)	24.75 ± 2.73 (17~30)	22.92 ± 3.58 (11~30)	1.64 ± 1.02 (0.5~5)	2.68 ± 1.19 (0.5~6)	4.39 ± 1.18 (1.5~7)

### Image acquisition

The datasets included standard T1-weighted MR images acquired from 1.5 T scanners using volumetric 3D MPRAGE (magnetization prepared rapid gradient echo) with a 1.25 × 1.25-mm in-plane spatial resolution and 1.2-mm thick sagittal slices, and the pixel resolution was 256 × 256. The data were collected from a variety of scanners using the protocol specified on the ADNI website.

### Data processing and cortical features computation

The images were processed and analyzed by the software FreeSurfer version 5.0.0 (http://surfer.nmr.mgh.harvard.edu/), which provides a set of tools that can be used for the analysis and visualization of structural and functional brain images. The FreeSurfer pipeline can produce regional cortical thickness and volumetric measurements including the local curvature, surface area, and the surface normal. First, image intensity variations due to magnetic fiagn inhomogeneities were corrected, and a normalized intensity image was generated (Sled et al., 1998). Second, skull stripping based on a hybrid watershed–surface-deformation method was adopted to remove the non-brain tissue (Segonne et al., 2004). Third, the obtained image was segmented based on the geometric structure of the gray–white interface, resulting in classified voxels belonging to either the white matter or non-white matter. Fourth, a triangular tessellation scheme was employed to generate a mesh of triangular faces, which was followed by several processes such as smoothing, inflation, and correction for topological defects (Fischl et al., 2001; Segonne et al., 2007), and the gray–white interface and the pial surface were thus obtained. Finally, two types of morphological measurements at each vertex were computed; these included volumetric features (cortical thickness, surface area, and GM volume) and geometric features (sulcal depth and curvature). The cortical thickness was calculated as the distance between linked vertexes on the interface and the pial surface. The surface area at each vertex was defined as the sum of the area of the triangles touching that vertex on the pial surface. The GM volume at every vertex was determined as the sum of the volumes of the individual triangles that lay within the neighborhood of the vertex, while the volume of each triangle was computed as the product of its area and the thickness at the center of the triangle. The two curvature measures, namely, mean curvature and Gaussian curvature, were derived from the principal curvatures, which were the maximum and minimum values of the curvatures of the various normal planes at the vertex. The Gaussian curvature of a surface at a vertex was the product of the two principal curvatures, and the mean curvature was the average of these. The sign of the Gaussian curvature was used to characterize the surface; the mean curvature described the average degree of bending of the surface at the vertex. The cortical surface was divided into 68 distinct cortical regions of interest (ROIs) according to the Desikan–Killiany atlas (Desikan et al., 2006); 34 cortical ROIs were extracted in each of the individual hemispheres.

In this study, four cortical morphological features were selected for discriminative analysis: GM volume (GMV), surface area (SA), cortical thickness (CT), and mean curvature (MC). To eliminate the effects of outliers and allow the comparison of corresponding values from different datasets, all the feature data were normalized as z-scores.

### Sparsity-constrained dimensionality reduction

The least squares problems usually occur in signal processing, regression, and classification. Given the training set $\left\{{\text{x}}_{1},{\text{x}}_{2},\cdots \;,{\text{x}}_{n}\right\}\in R^{d}$ and the corresponding class labels $\left\{{\text{y}}_{1},{\text{y}}_{2},\cdots \;,{\text{y}}_{n}\right\}\in R^{c}$, the basic model of the least square regression is:


(1) $$\underset{\text{W}}{\min}\sum_{i=1}^{n}{\left\|W^{T}x_{i}-y_{i}\right\|}_{2}^{2}$$

where **W** denotes the parameter matrix of size *d* × *c*, and ||.|| is the Frobenius norm of the matrix.

Instead of the traditional *l*_2_-*norm*, the *l*_2, 1_*-norm* was introduced to solve the optimization problem as follows:


(2) $$\underset{\text{W}}{\min}\sum_{i=1}^{n}{\left\|W^{T}x_{i}-y_{i}\right\|}_{2}$$

Let *p*_*i, j*_ be an element of matrix *p*∈*R*^*m* × *n*^, and the *l*_2, 1_-*norm* is defined as:


(3) $${\left\|P\right\|}_{2-1}\sum_{i=1}^{n}\sqrt{\sum_{j=1}^{m}p_{i,j}^{2}}$$

The *l*_2, 1_-*norm* has the peculiar ability to suppress the effect of outliers, which is similar to the *l*_2_-*norm*, while maintaining the rotational invariance as a fundamental property of the Euclidean space with the *l*_2_-*norm* (Ding et al., 2006; Kwak, 2008).

If a regularization term R(**W**) is included in Equation, we obtain:


(4) $$\underset{\text{W}}{\min}\sum_{i=1}^{n}{\left\|W^{T}x_{i}-y_{i}\right\|}_{2}+\lambda R(W)$$

where λ∈[01] is a constant coefficient. R(**W**) is defined as:


(5) $$R(W)={\sum_{i=1}^{d}\left\|w^{i}\right\|}_{2}$$

Therefore, the model with the regularization term in Equation (4) is given by:


(6) $$\begin{array}{l} \underset{\text{W}}{\min}\;J(\text{W})\;=\;\sum_{i=1}^{n}{\left\|W^{\text{T}}x_{\text{i}}-y_{\text{i}}\right\|}_{\text{2}}+\;\lambda \text{R}(W)={\left\|X^{\text{T}}W-Y\right\|}_{2,1}\\ \;+\;\lambda {\left\|W\right\|}_{2,1} \end{array}$$

The last term in Equation (6) is actually a penalty or regularization term on the model parameters to enforce sparsity; it can be solved by an effective iterative algorithm (Nie et al., 2010).

This sparsity-constrained dimensionality reduction model is called as the SCDR method, and the measurements associated with very small coefficients in the matrix **W**—which approximately equals zero—such as the threshold ε can be eliminated as nonessential features.

### SVM classifier

The SVM has been widely used as a powerful methodology in non-linear classification, regression, function estimation, density estimation, and feature selection and is introduced within the context of statistical learning theory and structural risk minimization. In classification problems, an SVM constructs a hyperplane or set of hyperplanes in a high or infinite dimensional space in order to achieve a good separation of the hyperplane that has the largest distance to the nearest training data point of any class (so-called functional margin), which thus leads to the best generalization ability for the unseen data points. In this study, a linear SVM with a linear kernel was selected for the classification, and the LIBSVM toolbox was used to perform the classification task (https://www.csie.ntu.edu.tw/~cjlin/libsvm).

To achieve the best performance and explore the dominant features associated with core regions, the retained features filtered by the SCDR were sorted using the coefficients of the parameter matrix, which represented the importance of each feature in the classification task, and then the SVM added each feature one-by-one in order to evaluate their performance. Therefore, the features with the best performance were determined. Leave-one-out cross validation (LOOCV) was used in the SVM classification owing to the small sample size. To avoid optimistically biased estimates of performance that result from using the same cross-validation to set the values of the hyper-parameters of the model and performance estimation, the 10-fold nested cross validation was used in the inner loop.

## Results

### Classification performance

The SVM classification of the three groups was performed using the cortical features. The classification performance was reported using accuracy, sensitivity, and specificity. The criteria for determining the best feature dimension was that the SVM should achieve the best comprehensive performance that would consist of a balance between accuracy, sensitivity, and specificity. The primary measure considered was the accuracy, followed by the sensitivity, and then the specificity. Each subject had 68 baseline features and 68 longitudinal features at each time point (e.g., 12 and 24 months).

For the sMCI–NC comparison using the GM volume, the SCDR determined 52 baseline GM volumes as the dominant features. The SVM classifier achieved its best performance when the baseline GM volumes of 25, 16, and 30 features were selected for the sMCI–NC, cMCI–NC, and sMCI–cMCI comparisons, respectively. As shown in Table 2, the accuracies were 76.27, 82.14, and 83.72% for the three comparisons, respectively. When the longitudinal GM volumes in 12 months were considered, the highest accuracy increased to 85.59, 87.5, and 90.7%, and the highest performance increased to 88.14, 91.96, and 93.2%, while all the follow-up features were combined with the baseline features, and the sensitivity and specificity were also significantly improved accordingly.

**Table 2:** **Classification performance**.

**Features**	**sMCI-NC**					**cMCI-NC**					**sMCI-cMCI**					
	**Retained feature number**	**Best feature number**	**ACC (%)**	**SEN (%)**	**SPE (%)**	**Retained feature number**	**Best feature number**	**ACC (%)**	**SEN (%)**	**SPE (%)**	**Retained feature number**	**Best feature number**	**ACC (%)**	**SEN (%)**	**SPE (%)**	
GMV_bl	52	25	76.3	58.7	87.5	45	16	82.14	62.5	93.06	40	30	83.72	82.5	84.78	
GMV_bl + 12 m	70	34	85.6	78.26	90.28	78	28	87.5	77.5	93.06	75	37	90.7	90	91.3	r = 1/4, Thres = 1e-1, ITER_NUM = 12
GMV_bl + 12 m + 24 m	99	74	88.1	84.78	90.28	95	26	91.96	87.5	94.44	97	57	93.2	95	91.3	r = 1/4, Thres = 1e-1, ITER_NUM = 12
CT_bl	66	18	78	63.04	87.5	30	30	82.14	67.5	90.28	54	30	84.88	85	84.78	
CT_bl + 12 m	57	47	86.4	82.61	88.89	46	39	89.29	82.5	93.06	85	39	84.88	87.5	82.61	
CT_bl + 12 m + 24 m	102	61	**92.4**	**84.78**	**97.2**	102	58	**93.8**	**92.5**	**94.44**	78	52	**98.8**	**97.5**	**100**	
SA_bl	48	32	77.1	58.7	88.89	48	20	77.68	62.5	86.11	46	34	81.4	75	86.96	
SA_bl + 12 m	78	56	83.9	73.91	90.28	79	50	75.89	65	81.94	65	31	90.7	92.5	89.13	
SA_bl + 12 m + 24 m	100	49	83.9	73.9	90.28	93	53	85.71	70	94.44	89	41	95.35	90	100	
MC_bl	53	32	76.3	52.17	91.67	50	28	80.36	70	86.11	50	38	73.26	77.5	69.57	
MC_bl + 12 m	108	52	72.9	60.87	80.56	91	40	83.04	72.5	88.89	80	48	93.5	92.5	97.83	
MC_bl + 12 m + 24 m	95	61	89.8	86.96	91.67	86	86	91.07	87.5	93.06	70	65	96.51	97.5	95.65	

*GMV, gray matter volume; CT, cortical thickness; SA, surface area; MC, mean curvature; 12 m, 12 months; 24 m, 24 months; ACC, accuracy; SEN, sensitivity; SPE, specificity The bold values are the best performance*.

**Figure 1:**
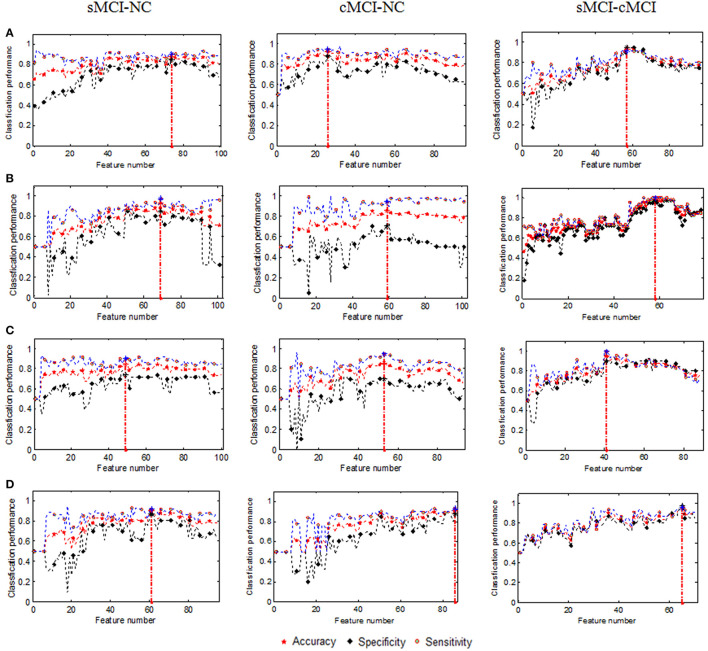
**Changes in classification performance with the increase in combined features (A)** GMV, **(B)** Cortical thickness, **(C)** Surface area, and **(D)** Mean curvature. The vertical red dot lines denoted the best performances.

The changes in performance of the SVM with the increasing dimensionality of the combined features are shown in Figure 1.

The number of retained GM volumes filtered by the SCDR was 99, 95, and 97 for the sMCI–NC, cMCI–NC, and sMCI–cMCI comparisons, respectively. As shown in Figure 1A, the sorted GM volumes were added into the SVM classifier one by one. The accuracy, sensitivity, and specificity fluctuated and then reached the peak with 88.14% accuracy, 84.78% sensitivity, and 90.28% specificity in the sMCI–NC classification using 74 features; 91.96% accuracy, 87.50% sensitivity, and 94.44% specificity in the cMCI–NC classification using 26 features; and 93.20% accuracy, 95.00% sensitivity, and 91.30% specificity in the sMCI–cMCI classification using 57 features. However, with the increase in the features, the performances degraded gradually. Therefore, the use of all the retained features may not ensure the best performance of the SVM classifier.

**Figure 2:**
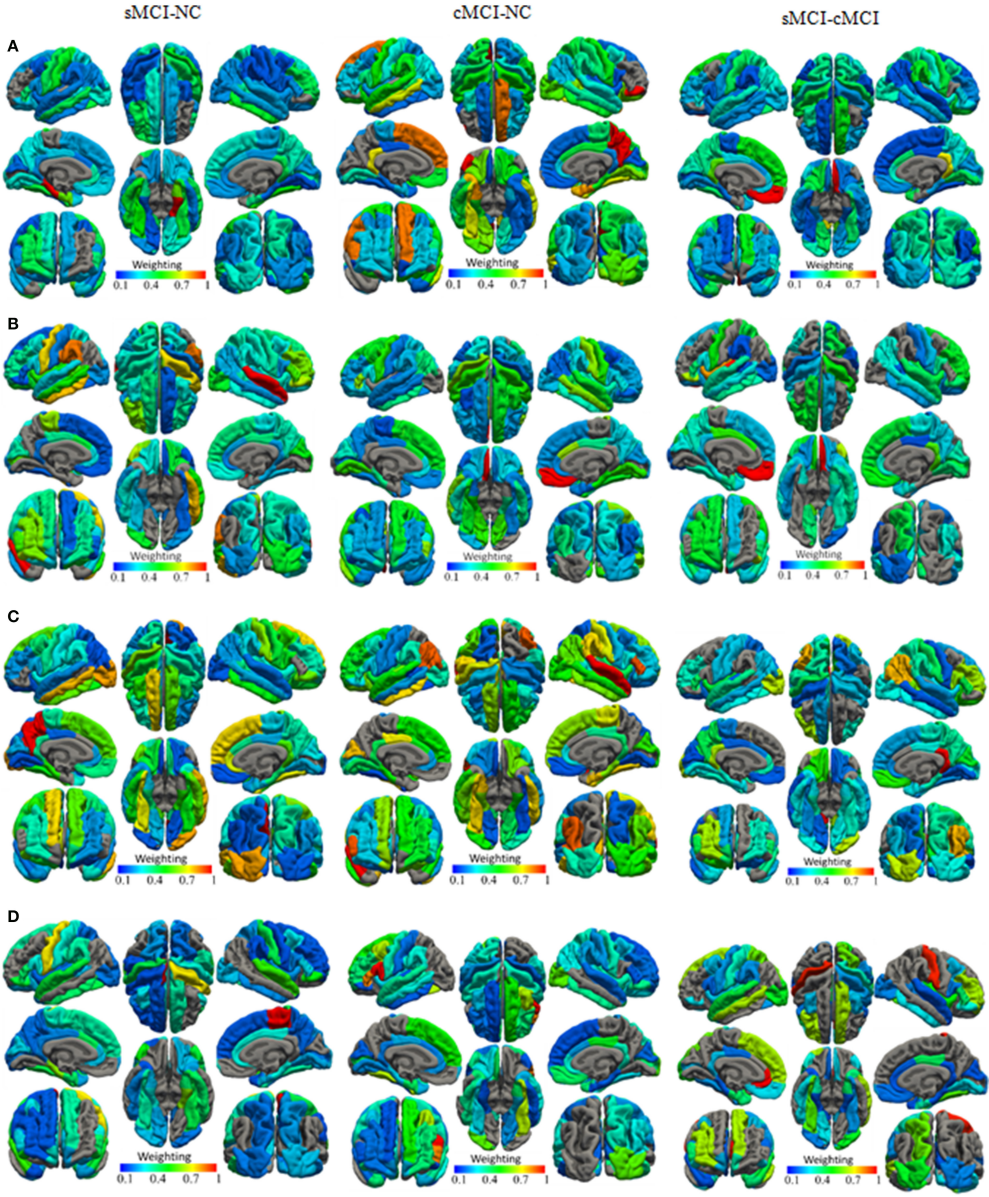
**The normalized weights of combined cortical surface features with the highest (A)** GM volume, **(B)** Cortical thickness, **(C)** Surface area, and **(D)** Mean curvature.

**Figure 3:**
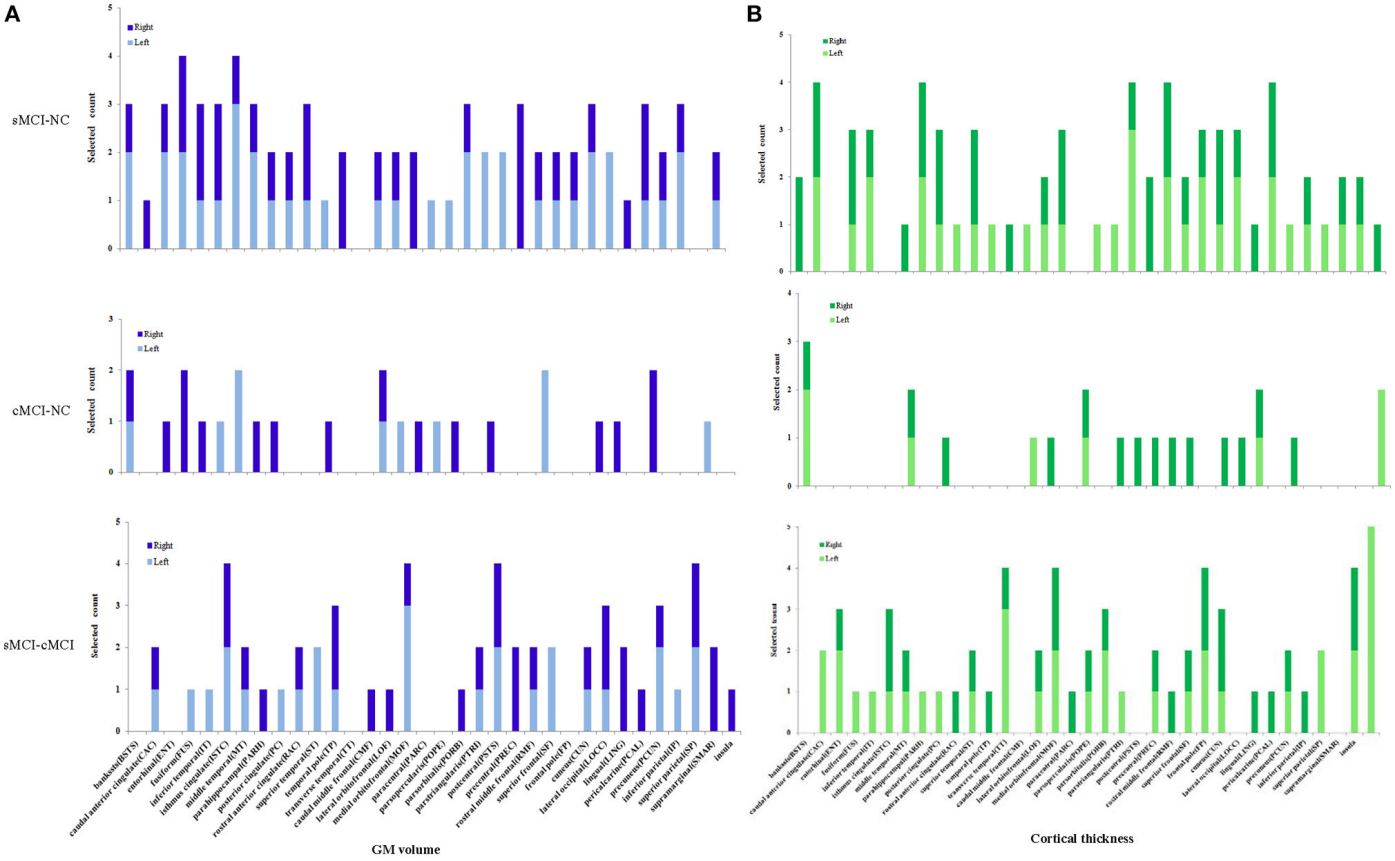
**Selected count of GM volume and cortical thickness in 68 brain regions**. Selected count means the count of combined features were selected for classification with the high performance, left, right denotes left and right hemisphere. **(A)** GM volume. **(B)** Cortical thickness.

**Figure 4:**
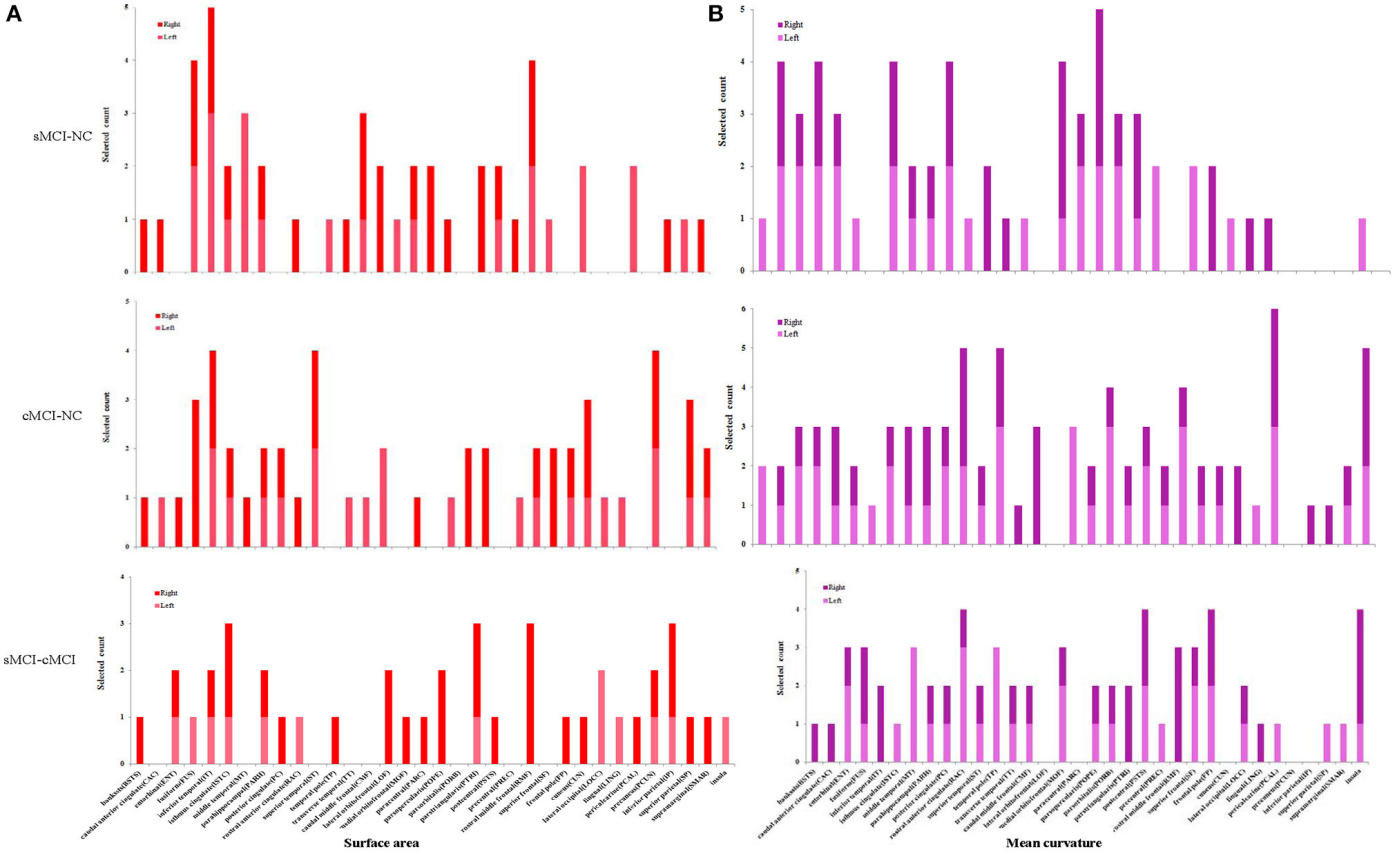
**Selected count of surface and mean curvature in 68 brain regions. (A)** Surface area. **(B)** Mean curvature.

With regard to the normalized weights and the spatial distribution of the selected features from the baseline measures combined with the longitudinal measures, The selected cortical surface features with the highest performance were mapped onto the surface of the brain as shown in Figures 2–**4** listed the selected count of the features in 68 brain regions. It revealed different patterns of the GM volume and its underlying changes between the compared groups. For example, in the sMCI–NC comparison, most of the brain regions were involved in the classification, which revealed that sMCI patients suffered from atrophies in many cortexes and especially in the temporal lobe. For the sMCI–cMCI comparison, the brain regions included retained 57 features also were located in all lobes. Although only 26 features from 22 regions were determined, they had a prominent ability to discriminate between the cMCI and the NC groups, and most of the associated regions were in the right hemisphere.

Similarly, the baseline cortical thickness and the longitudinal changes were adopted for discrimination using the SVM. The accuracies were 77.97, 86.44, and 92.37% for the sMCI–NC, cMCI–NC, and sMCI–cMCI comparisons, respectively, using the baseline cortical thickness. The performance was improved significantly when the longitudinal features of the cortical thickness in 12 months were combined, and the accuracies reached 82.14, 89.29, and 93.75% for the three comparisons, respectively. The changes in classification performance with the increase of the combined cortical thickness were shown in Figure 1B. The SVM achieved the highest accuracy (92.37, 93.75, and 98.84%) using the combined measures from all time points; a similar result was obtained for the sensitivity and specificity.

For the surface area and mean curvature, the three groups were discriminated using a similar pattern with the GM volume and the cortical thickness using the baseline and dynamic features as listed in Figures 1, 3, 4 and Table 2.

Using the selected baseline features, the SVM produced poor accuracies ranging from 73 to 85%, low sensitivities ranging from 52 to 85%, and low specificities ranging from 69 to 93% for all the comparisons. However, the classifier performed better using the selected baseline and longitudinal features in 12 months with accuracies of 72–94%, sensitivities of 60–92.5%, and specificities of 80–98%, and it achieved the highest performance with accuracies of 83–99%, sensitivities of 70–98%, and specificities of 90–100%. Generally, the cortical thickness provided the best performance in the between-group classification among the NC, sMCI, and cMCI groups, followed by the GM volume and the mean curvature, while the surface area had the weakest ability for classification. Based on the selected features of all the time points, the cortical thickness in particular exhibited prominent discriminative power with an accuracy, a sensitivity, and specificity of 92.37, 84.78, and 97.22% respectively between the sMCI and the NC groups; 93.7, 92.5, and 94.44% respectively in the cMCI–NC comparison; and 98.84, 97.5, and 100% respectively in the cMCI–sMCI comparison.

### Spatial distribution of the essential features

According to the spatial distribution of the selected features, it covered most of the cortex in the temporal, frontal, occipital, and parietal lobes and insula, which revealed that there existed significant morphological differences between the three groups. As is well known, global brain atrophy has been observed in the healthy elderly and patients with MCI, and it varied in the regional atrophy pattern between the three groups, including the atrophy speed or rate, the brain region distribution (Shiino et al., 2006; Li et al., 2012; Clerx et al., 2013; Eskildsen et al., 2013a; Lampert et al., 2014), which were the basis for the discrimination. Furthermore, there were significant differences in both feature number and spatial distribution for different cortical features and comparisons. In order to elucidate our findings, we focus on the results of the combined features at all-time points. For the sMCI–NC comparison using GM volume, it was found that the GM volume in most brain regions provided effective information. Only seven regions in the left hemisphere and eight regions in the right hemisphere were excluded: the left caudal anterior cingulate, transverse temporal, caudal middle frontal, paracentral, rostral middle frontal, rostral middle frontal, pericalcarine, and supramarginal; and right temporal pole, caudal middle frontal, parsopercularis, parsorbitalis, postcentral, precentral, lingual, and supramarginal. For the cMCI–NC comparison, 21 regions were involved in the classification: 7 regions in the left temporal and frontal lobes and 14 regions in the temporal, frontal, and occipital lobes. It was suggested that the GM volume from these 22 regions provided sufficient information to distinguish the cMCI from the NC subjects. The underlying reason was that the GM volume decreased more sharply in these regions than the NC group; the cMCI more closely resembled the AD in terms of topography of the GM volume decrease and cortical thinning than the sMCI (Ye et al., 2014). It was noticed that the selected features covered most of the regions in the comparison between the sMCI and the cMCI, which was ascribed to higher amyloid deposition in the case of the latter (Landau et al., 2010, 2012). The spatial distribution of the selected cortical thickness measurements was quite similar to that of the GM volume. Twenty-six left regions and 24 right regions from all lobes were selected for the sMCI–NC comparison, 7 left regions and 14 right regions were effective for the sMCI–NC classification, and 22 left regions and 22 right regions that covered most of the cortex were involved in the discrimination of two types of MCI.

The other two measures, the surface area and the mean curvature, had patterns similar to the GM volume and the cortical thickness for the sMCI–NC (with accuracies of 77–84% and 76–90% respectively), sMCI–cMCI classification (with accuracies of 81–96% and 73–97% respectively), and the cMCI–NC classification (with accuracies of 77–85% and 80–92% respectively). However, more features and brain regions were included for the cMCI–NC classification than that using the GM volume and the cortical thickness.

## Discussion

A sparsity-constrained dimensionality reduction model was applied to select the essential features for discriminative analysis on brain cortical measurements between the sMCI group and the cMCI group. Four types of measurements including cortical thickness, surface area, mean curvature, and GM volume were investigated from both baseline and longitudinal MR images. Our results suggested that the cortical thickness had the strongest power to discriminate for the between-group classification, followed by the GM volume, the mean curvature, and the surface area. The longitudinal changes in the cortical measurements could provide effective information for discrimination and improved the performance of the classifier.

It was observed that the retained features filtered by the SCDR did not have the best discriminative capability in most of the classifications. The performance of the SVM classifier using all the retained features was 5–40% less than that using the subset of the features with the strongest discriminative ability. This was attributed to the optimized strategy of feature adding in turn by the ordered weight of each feature acquired using the SCDR, which represented its importance or capability in discrimination.

With respect to the relationship between the selected regional distribution and the neuropsychological dysfunction, it was speculated that the two MCI groups had atrophy or cortical thinning not only in early affected regions such as in the lateral temporal cortex and frontal cortex, but also in the parietal cortex, occipital cortex, and insula at a relatively late stage. This was in agreement with the findings due to regional amyloid deposition in previous studies (Bouwman et al., 2007; Cui et al., 2011; Camus et al., 2012). As an intermediate stage between the expected cognitive decline of normal aging and the more serious decline of dementia, the patients with MCI had impairments in memory, language, thinking, and judgment. It was remarkable that the cingulate regions and insula played a key role in both the cMCI–NC classification and the sMCI–cMCI classification, which included the caudal anterior cingulate, isthmus cingulate, posterior cingulate, rostral anterior cingulate, and insular cortex. The cingulate cortex, a part of the limbic cortex, is linked to emotion formation and processing, learning, and memory, while the insula is believed to be linked to diverse functions such as perception, motor control, self-awareness, cognitive functioning, and interpersonal experience. However, very few regions were retrieved for the cMCI–NC classification when either the GM volume or the cortical thickness was considered; these regions included the middle temporal, posterior cingulate, lateral orbitofrontal, postcentral, and lateral occipital cortex, and they provided strong discriminative power that was enough to distinguish the cMCI from the NC. These regions are highly correlated with decision making, sense, recognition, and vision; the posterior cingulate cortex, especially, formed a central node in the default mode network of the brain, which was demonstrated to communicate with various brain networks simultaneously and was involved in various functions. Moreover, there was bilateral asymmetry; more regions in the right hemisphere were retrieved than those in the left hemisphere in the cMCI–NC comparison.

Furthermore, the selected regional distribution of two MCI groups illustrated that most of brain regions were involved in classification, especially those regions with strong discriminative ability were isthmus cingulate, temporal pole, transverse temporal in temporal lobe, rostral middle frontal, frontal pole in frontal lobe and insula. As well known, isthmus cingulate was connected by a narrow isthmus with the parahippocampal gyrus, the temporal lobe was a particularly complex brain area involved in a diversity of functions including auditory, olfactory, memory, vestibular, visual, and linguistic processing, transverse temporal participated in processing incoming auditory information. The rostral middle frontal gyrus, was critical for executive function, including emotion regulation and working memory. The frontal pole was closely related to retrospective memory and could effectively guide goal-directed behavior. As mentioned above, the insula was believed to be involved in consciousness and emotional functions. These finding revealed that the cMCI group suffered from cognitive dysfunction in relation to diverse tasks including auditory, olfactory, memory, visual, linguistic and emotional processing. In comparison with the sMCI group, the cMCI group experienced greater atrophy or thinning in these cortexes, which facilitated progression of cognitive dysfunction and conversion to dementia.

The three parameters including the constant coefficient λ, the iterations, and the threshold ε of the SCDR model, play an essential role in dimension reduction. λ controls the strength of the sparse constraint, a large value of λ will lead to a large decrease in feature dimension and accelerate the convergence, and vice versa. The iterations determine the repetition times to obtain the final resolution. The threshold ε performs as a filter to select the salient features corresponding to the coefficients with a weight greater than the threshold and eliminates the insignificant features. λ, ε, and the iterations were set to 0.25, 0.01, and 15 respectively in this work.

There are several limitations associated with our study. First, the correlation, and importance of the features were implicit (unlike in statistical testing), and thus, the significant differences between the compared groups were found, and the discriminative power of the extracted measures was evaluated. However, the correlations between the core features, changes of these features, and cognitive impairment measurements remained undetermined. Second, only the cortical structural information was explored; the correlation between the cortical features with other types of measurements including neuroimaging data such as functional MRI, DTI, MRS, genetic and chemical biomarkers remain to be discovered, and there is great scope for further development by combining them for effective classification and conversion prediction. Third, the number of subjects was limited to 158, and only 2-year follow-up studies were investigated. The information obtained would have been more valuable if a larger sample size and longer period had been considered.

## Conclusion

This study investigated four cortical surface features to discriminate two types of patients with sMCI and cMCI, from the NC using 2 years of follow-up MR images. A sparse-constrained model was presented to reduce the feature dimensionality in order to retrieve the core measurements and relative brain regions for effective discrimination. The performance of the SVM was significantly improved when the baseline cortical features were combined with the longitudinal features, which could effectively reveal morphological changes such as cortical thinning or GM atrophy. It was highlighted that the SCDR could not only eliminate redundant and non-significant features, but also determine the weights to indicate the importance of the essential features. Consequently, the best performance could be achieved using the strategy of feature adding in turn based on their weights.

It was demonstrated that the sparse-constrained model could determine vital information from cortical measurements for effective classification between the three compared groups. Our results suggest that it is feasible to distinguish the sMCI and the cMCI from healthy elderly using static cortical features combined with dynamic measurements, and this proposed method has great potential in monitoring the advancement of MCI in patients and predicting the conversion of MCI to AD.

## Ethics statement

Data used in this study were obtained from the Alzheimer's Disease Neuroimaging Initiative (ADNI) (http://adni.loni.usc.edu/). The ADNI data were previously collected across 50 research sites. Study subjects gave written informed consent at the time of enrollment for imaging and genetic sample collection and completed questionnaires approved by each participating sites' Institutional Review Board (IRB). All procedures performed in studies involving human participants were in accordance with the ethical standards of the institutional and/or national research committee and with the 1964 Helsinki declaration and its later amendments or comparable ethical standards.

## Author contributions

SG: Design of the work and write the manuscript. CL: Implement the algorithm. CW and GC: Obtain results. All authors approves the final version of the work and agree to be accountable for all aspects of the work in ensuring that questions related to the accuracy or integrity of any part of the work are appropriately investigated and resolved.

## Funding

This study was partly supported by the National Natural Science Foundation of China (31371008), Science and Technology Planning Project of Guangdong Province (2015A02024006), Guangzhou Bureau of Science and Technology (201604020170) and the China Scholar Council.

### Conflict of interest statement

The authors declare that the research was conducted in the absence of any commercial or financial relationships that could be construed as a potential conflict of interest.
