# Corrigendum: Conversion Discriminative Analysis on Mild Cognitive Impairment Using Multiple Cortical Features from MR Images

**DOI:** 10.3389/fnagi.2017.00293

**Published:** 2017-09-05

**Authors:** Shengwen Guo, Chunren Lai, Congling Wu, Guiyin Cen, The Alzheimer's Disease Neuroimaging Initiative, Michael W. Weiner

**Affiliations:** Author Affiliations: UC San Francisco; University of Southern California; UC San Francisco University of Southern California Mayo Clinic, Rochester Mayo Clinic, Rochester; UC Berkeley; U Pennsylvania; USC; UC Davis; Brigham and Women's Hospital/Harvard Medical School Indiana University Washington University St. Louis University of Pennsylvania; Prevent Alzheimer's Disease 2020 (Chair) Siemens; Alzheimer's Association University of Pittsburgh Washington University St. Louis Cornell University; Albert Einstein College of Medicine of Yeshiva University; AD Drug Discovery Foundation; Acumen Pharmaceuticals; Washington University St. Louis; Northwestern University; National Institute of Mental Health; Brown University; Eli Lilly (Chair); BWH/HMS (Chair); University of Washington (Chair); Mayo Clinic, Rochester (Core PI) University of Southern California; UC San Diego; UC San Diego; UC San Diego; UC San Diego; UC San Diego; UC San Diego; UC San Diego; UC San Diego; UC San Diego; UC Davis (Core PI); UC Davis; UC San Diego; Mayo Clinic, Rochester (Core PI); Mayo Clinic, Rochester; University of London; UCLA School of Medicine; UCSF MRI; UC Davis; Mayo Clinic; Mayo Clinic; Mayo Clinic; Mayo Clinic; Mayo Clinic; Mayo Clinic; Mayo Clinic; UC Berkeley (Core PI); University of Michigan; University of Utah; Banner Alzheimer's Institute; Banner Alzheimer's Institute; University of Pittsburgh; UC Berkeley; Washington University St. Louis; Washington University St. Louis; Washington University St. Louis; Washington University St. Louis; UPenn School of Medicine; UPenn School of Medicine; UPenn School of Medicine; UPenn School of Medicine; UPenn School of Medicine; USC (Core PI); USC; USC; Indiana University; Indiana University; UC Irvine; Indiana University; Indiana University; Indiana University; Indiana University; UC San Francisco; UC San Diego; Prevent Alzheimer's Disease 2020; UC San Diego; National Institute on Aging; UC San Francisco; Brown University; National Institute of Mental Health; Cornell University; Johns Hopkins University; Richard Frank Consulting; Prevent Alzheimer's Disease 2020; National Institute on Aging; Oregon Health & Science University; University of Southern California; University of California - San Diego; University of Michigan; Mayo Clinic, Rochester; Baylor College of Medicine; Columbia University Medical Center; Washington University, St. Louis; University of Alabama - Birmingham; Mount Sinai School of Medicine; Rush University Medical Center; Wien Center; Johns Hopkins University; New York University; Duke University Medical Center; University of Pennsylvania; University of Kentucky; University of Pittsburgh; University of Rochester Medical Center; University of California, Irvine; University of Texas Southwestern Medical School; Emory University; University of Kansas, Medical Center; University of California, Los Angeles; Mayo Clinic, Jacksonville; Indiana University; Yale University School of Medicine; McGill Univ., Montreal-Jewish General Hospital; Sunnybrook Health Sciences, Ontario; U.B.C. Clinic for AD & Related Disorders; Cognitive Neurology - St. Joseph's, Ontario; Cleveland Clinic Lou Ruvo Center for Brain Health; Northwestern University; Premiere Research Inst (Palm Beach Neurology); Georgetown University Medical Center; Brigham and Women's Hospital; Stanford University; Banner Sun Health Research Institute; Boston University; Howard University; Case Western Reserve University; University of California, Davis - Sacramento; Neurological Care of CNY; Parkwood Hospital; University of Wisconsin; University of California, Irvine - BIC; Banner Alzheimer's Institute; Dent Neurologic Institute; Ohio State University; Albany Medical College; Hartford Hospital, Olin Neuropsychiatry Research Center; Dartmouth-Hitchcock Medical Center; Wake Forest University Health Sciences; Rhode Island Hospital; Butler Hospital; UC San Francisco; Medical University South Carolina; St. Joseph's Health Care; Nathan Kline Institute; University of Iowa College of Medicine; Cornell University; University of South Florida: USF Health Byrd Alzheimer's Institute; University of California, San Francisco; University of Southern California; UC San Francisco; University of Southern California; Mayo Clinic, Rochester; Brigham and Women's Hospital/ Harvard Medical School; UC Davis; Mayo Clinic, Rochester; UC Berkeley; Washington University St. Louis; Indiana University; Perelman School of Medicine, UPenn; USC; Perelman School of Medicine, University of Pennsylvania; UC San Francisco; Rehabilitation Institute of Chicago, Feinberg School of Medicine, Northwestern University; BWH/HMS (Chair); University of Washington (Chair); Core PI; Mayo Clinic, Rochester (Core PI); University of Southern California; UC San Diego; UC San Diego; UC San Diego; UC San Diego; UC San Diego; UC San Diego; UC San Diego; UC San Francisco; UC San Francisco; UC San Francisco; UC Davis (Core PI); UC San Diego; Mayo Clinic, Rochester (Core PI); Mayo Clinic, Rochester; Mayo Clinic; Mayo Clinic; Mayo Clinic; Mayo Clinic; Mayo Clinic; UC Berkeley (Core PI); University of Michigan; University of Utah; Banner Alzheimer's Institute; Banner Alzheimer's Institute; UC Berkeley; Washington University St. Louis; Washington University St. Louis; Washington University St. Louis; Perelman School of Medicine, UPenn; Perelman School of Medicine, UPenn; Perelman School of Medicine, UPenn; Perelman School of Medicine, UPenn; Perelman School of Medicine, UPenn; USC (Core PI); USC; USC; Indiana University; Indiana University; UC Irvine; Indiana University; Indiana University; Indiana University; Indiana University; UC San Francisco; Department of Defense (retired); University of Southern California; University of California, San Diego; Columbia University Medical Center; Rush University Medical Center; Wien Center; Duke University Medical Center; University of Rochester Medical Center; University of California, Irvine; Medical University South Carolina; Premiere Research Inst (Palm Beach Neurology); University of California, San Francisco; Georgetown University Medical Center; Brigham and Women's Hospital; Banner Sun Health Research Institute; Howard University; University of Wisconsin; University of Washington; Stanford University; Cornell University; ^1^Department of Biomedical Engineering, South China University of Technology Guangzhou, China; ^2^Guangdong General Hospital Guangzhou, China

**Keywords:** mild cognitive impairment, conversion, cortical feature, sparse-constrained regression, feature reduction, classification

Due to an oversight, the link used to list authors and grant authorship to Alzheimer's Disease Neuroimaging Initiative (ADNI) members was incorrect. The correct link is appearing below:

http://adni.loni.usc.edu/wp-content/uploads/how_to_apply/ADNI_Acknowledgement_List.pdf

Authorship of ADNI members has been corrected.

The authors apologize for this error and state that this does not change the scientific conclusions of the article in any way.

The original article has been updated.

## Conflict of interest statement

The authors declare that the research was conducted in the absence of any commercial or financial relationships that could be construed as a potential conflict of interest.

